# Clinicopathological Evaluation of Acute Leukemias in a Tertiary Care Hospital: A Cross-Sectional Study

**DOI:** 10.5146/tjpath.2021.01524

**Published:** 2021-05-15

**Authors:** Gayatri N. Patel, Rashmi Gudur, Anand Gudur, R. M. Oswal, Sujatha Kanethkar

**Affiliations:** Department of Pathology, Krishna Institute of Medical Sciences (Deemed to University), Maharashtra, India; Department of Oncology, Krishna Institute of Medical Sciences (Deemed to University), Maharashtra, India

**Keywords:** Acute myeloid leukemia, WHO classification 2016, Flow cytometry, Cytogenetics, Molecular study

## Abstract

***Objective:*** Acute Myeloid Leukemia (AML) and Acute Lymphoblastic Leukemia (ALL) are clinically and biologically diverse phenotypic diseases amongst hematological malignancies. The current study objectives were to diagnose and classify cases of AL as per revised 4th edition of WHO 2016 classification of AL’s and study their clinicopathological profiles.

***Material and Method:*** This cross-sectional, observational study included 68 patients, diagnosed with AL were recruited. Diagnosis was based on peripheral blood smear examination, bone marrow aspiration, flowcytometry, and cytogenetic and molecular studies.

***Results:*** Sixty-eight cases of AL were diagnosed in a period of 2 years, where 25 cases were of ALL and 43 cases were of AML. In the subclassification of AML as per WHO 2016, 20 cases were of AML, RGA, 21 cases were of AML, NOS, and 2 cases were of AML, MRC. In AML, RGA, APL with PML-RARA positive cases were 10 out of 20 cases, AML with (8;21) RUNX1-RUNX1T1 were 7/20 cases; there were two cases of AML with mutated NPM1 gene and one case of AML with biallelic mutation of CEBPA. In AML, NOS subcategory AML with maturation was more common with 9/21cases. In subcategory of ALL, B-ALL was more common than T-ALL. B-ALL, NOS was more common than B-ALL, RGA and we had 1 case of NK cell Leukemia.

***Conclusion:*** The application of revised 4th edition WHO 2016 classification confers uniformity in reporting acute leukemia cases that aids in the treatment by using targeted therapies and helps in the prediction of prognosis. The WHO classification for acute leukemias is very objective, therapy oriented and the need of the hour.

## INTRODUCTION

Acute leukemias (AL) constitute a heterogeneous group of hematological malignancies, characterized by an uncontrolled proliferation of hematopoietic cells that infiltrate into the bone marrow and blood (1).

Acute myeloid leukemia (AML) and acute lymphoblastic leukemia (ALL) are the two main types of AL, with a global prevalence of 3-4 and 0.4-2 per 100,000 individuals, respectively (2–4). The annual incidence in India is estimated to be 4.3 and 1.4 per 100,000 individuals for AML and ALL, respectively (5,6). AML is more prevalent in adults and ALL in children (7).

The FAB classification system was proposed in 1976 for classifying these diseases in order to treat them as per their biologic behavior. However, it had its own set of drawbacks as it was based on morphology alone and led to subjective errors (8). This subjectivity in diagnosis was eliminated by the WHO classification system, proposed in 1999 and revised in 2001, 2008, and 2016 (published in 2017). The WHO (2016) classification system was evolved to improve the objectivity and reproducibility by additionally utilizing the cytogenetic, molecular, cytochemical, and immunologic characteristics for an integrated diagnosis (9,10). This has greatly contributed to the determination of differential diagnosis and prognosis of leukemic proliferations, facilitating targeted treatment as per their pathologic behavior. This includes management of recognizable genetic lesions, stem cell transplantation as well as immunotherapy such as treatment directed at specific cluster differentiation (CD) markers (11). The regular revisions of AL classifications have given rise to a constant need to study these pathologies in order to upgrade the management strategies.

The present study aims to diagnose and classify AL cases according to the WHO Classification of Tumors of Hematopoietic and Lymphoid Tissues, 2016, revised 4th edition and study their clinicopathological profiles (9).

## MATERIALS and METHODS

This cross-sectional, observational study was conducted at the Department of Pathology, in a tertiary teaching hospital in India, from June 2017 to May 2019, after obtaining ethical clearance from the Institutional Review Board (protocol number: 031/2017-18, Date: 23.11.2017).

In all, 68 patients diagnosed with AL (AML and ALL) as per the WHO Classification of Tumors of Hematopoietic and Lymphoid Tissues, 2016, revised 4th edition criteria, irrespective of age and gender, were recruited into the study after obtaining written informed consent from them (or their legal guardians in case of minor patients) (6). AL cases without cytogenetic studies or advanced ancillary techniques were excluded from the study. Patients were examined for pallor, fever, generalized weakness, bony tenderness, petechiae, ecchymosis, and gum bleeding. Presence of hepatomegaly, splenomegaly, and lymphadenopathy was also recorded. Ultrasonography (USG) was performed where necessary. Morphologic dysplasia, cytogenetic abnormalities, and discontinuation of chemotherapy due to low blood cell count were considered as unfavorable prognostic factors.

### Hematological Investigation

This included estimation of hemoglobin (Hb) levels, total leukocyte count (TLC), differential leukocyte count (DLC), and platelet count, using the Sysmex XT-1800i (XT-1800i, Sysmex Corporation, Kobe, Japan). Peripheral venous blood samples were collected in ethylenediaminetetraacetic acid (EDTA)-anticoagulated vacutainers. Peripheral smears were made, stained with the Romanowsky Leishman stain, and studied in detail for morphology. In cases of leukopenia, buffy-coat smears were prepared and stained with the Leishman stain and examined for the presence of blast cells. A provisional diagnosis was made and ancillary studies performed.

### Immunophenotyping

All cases underwent flow-cytometric analyses using BD FACSCanto™II/FACSDiva™ 6-color flowcytometry (BD Biosciences, Becton, Dickinson, and Company, New Jersey, USA), molecular studies using nested/reverse transcriptase polymerase chain reaction (PCR), and cytogenetic studies using fluorescence in-situ hybridization (FISH).

### Bone Marrow Aspiration Study

This was performed on all patients under aseptic conditions and local infiltration anesthesia with 3 cc of 2% lignocaine. Bone marrow aspiration was performed from the posterior superior iliac spine using Salah’s aspiration needle. Smears were prepared, air dried, and stained with the Giemsa stain. If the marrow particles were not visibly present in the aspirate, it was centrifuged and the smears prepared accordingly. The smears were meticulously examined for cellularity, predominant series, myeloid:erythroid ratio, erythroid series cells, myeloid series cells, lymphocytes, plasma cells, megakaryocytes, blast cells, atypical cells, mitosis, iron stores, and parasites. At least 500 cells were counted to obtain the myelogram.

### Bone Marrow Trephine Biopsy

Indicated only when a dry tap was obtained or the aspirate was inadequate for diagnosis (6 out of 68 cases). Bone marrow was obtained from the posterior superior iliac spine using a Jamshidi™ needle (BD Biosciences, Becton, Dickinson, and Company, New Jersey, USA) under aseptic conditions and local infiltration anesthesia with 2% lignocaine. The biopsy needle was introduced in the bone cortex with a rotatory movement and gently advanced after removing the stylet. An optimal 1-2 cm long biopsy sample was obtained in its maximum diameter, placed in 10% formalin fixative, decalcified in 14% EDTA solution, submitted for routine paraffin processing, and stained with hematoxylin and eosin for further examination.

### Statistical Analysis

The data was collected, compiled, and analyzed using SPSS version 20 software (IBM Corp., released 2011, IBM SPSS Statistics for Windows, Version 20.0. Armonk, NY: IBM Corp.). Categorical variables were expressed in terms of frequencies and percentages.

## RESULTS

**Figure 1:**
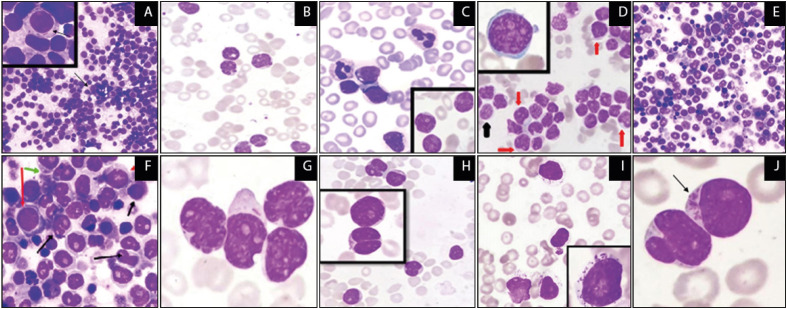
**A)** Hypercellularity in BMA with blast cells, Arrow shows blast cell with Auer rod in AML. **B)** PBS Showing large blasts with variable N:C ratio and scant-to-moderate amount of basophilic cytoplasm, absence of maturing granulocytes indicate AML-NOS. **C)** PBS showing large blasts features with matured nuclei favoring AML-NOS with maturation. **D)** PBS Showing severe leukocytosis with blasts features favoring AML-NOS (Black arrow showing Monoblasts ; Red arrow showing Promonocytes). **E)** BMA showing case of AMLNOS. **F)** BMA of AML-NOS showing neutrophilic precursors (Black arrow showing myelocytes ; Red arrow showing Promonocytes; green arrow shows auer rod myeloblast). **G)** PBS of APL showing bilobed or buttock-shaped nuclei with moderate cytoplasm. **H)** PBS of A case of APL with PML-RARA. **I)** PBS showing large sized lymphocytes and azurophilic granules indicating PBP-NKLL. **J)** PBS showing Multiple Auer rods cells in criss-cross pattern indicating AML. (All the images shown are x400; inserts are x1000 ; BMA and PBS samples were stained by Giemsa and Leishman stains, respectively). ***AML:*** *Acute myeloid leukemia,* ***APL:*** *Acute promyelocytic leukemia,* ***BMA:*** *Bone marrow aspirate,* ***NOS:*** *Not otherwise specified,* ***PBS:*** *Peripheral blood smear,* ***s/o:*** *Suggestive of*

The present study included 68 cases of AL, of which 43 (63.24%) were diagnosed with AML and 25 (36.76%) with ALL. Covering an age range of 10-70 years, the mean age of the included participants was 37.07±21.15 years, with a slight male predominance (M:F = 1.7:1). The average age was 48.16±16.18 years for the AML cases and 8.28±13.85 years for the ALL cases. Both were more common in males (M:F = 1.5:1 in AML; 2.1:1 in ALL). The frequency distribution of different types of AL is described in Table 1. The frequency distribution of the clinical features of AL is shown in Table 2. Fever was the most common presenting symptom (91.81%) followed by generalized weakness (80.88%). The most common presenting sign was pallor (94.19%) followed by splenomegaly (80.88%). Table 3 summarizes the hematological characteristics of these subjects. Anemia (Hb 7.2 g%), leukocytosis (>100,000/mm3), and thrombocytopenia (<40,000mm3) were seen in all the cases, with a low neutrophil count (7.6%) and an increased peripheral blast cell count of >20% (reaching an average of 63%). Granulocyte precursor cells like myelocytes, metamyelocytes, promyelocytes, and promonocytes were increased in cases of AML. The immunophenotyping findings in these AL cases are detailed in Table 4. Table 5 presents the distribution of AL cases according to their prognosis. The comparative results revealed that the prognosis was more on the unfavorable side for all the leukemia types, and all the patients having unfavorable prognosis succumbed to their illness within 6-9 months after diagnosis. The histological findings from the peripheral smear and bone marrow aspirate are presented in Figure 1.

**Table 1:** Frequency distribution of different types of acute leukemias

**Type of Acute Leukemia**	**No. of subjects**	**Percentage (%)**
**ALL**	25	36.76
**1) T-ALL**	6	76
1a) T-ALL NOS	5	83.3
1b) T-ALL NK	1	16.7
**2) B-ALL**	19	24
2a) B-ALL NOS	15	78.9
2b) B-ALL RGA (i) B-ALL with t(12;21)(p13;q22)-TEL AML1(ETV6 RUNX1) (ii) B-ALL with t(9;22)(q34;q11.2)-BCR ABL1	4 3 1	21.1
**AML**	43	63.24
**1) AML - RGA**	20	46.51
1a) AML with t(8;21)(q22;q22.1) RUNX1 RUNX1T1	7	
1b) APL with PML RARA	10	
1c) AML with mutated NPM1	2	
1d) AML with biallelic mutations of CEBPA	1	
**2) AML – MRC**	2	4.66
**3) AML – NOS**	21	48.83
3a) AML with minimal differentiation	2	
3b) AML without maturation	2	
3c) AML with maturation	9	
3d) Acute myelomonocytic leukemia	3	
3e) Acute monoblastic leukemia	4	
3f) Pure erythroid leukemia	1	

**AML:** Acute myeloid leukemia, **ALL:** Acute lymphoblastic leukemia, **NOS:** Not otherwise specified, **NK:** Natural killer cell, **RGA:** Recurrent genetic abnormality, **MRC:** Myelodysplasia-related changes, **APL:** Acute promyelocytic leukemia.

**Table 2:** Frequency distribution of clinical features of acute leukemias

**Clinical features**	**AML (n=43)**	**ALL (n=25)**	**Total (n=68)**	**Percentage (%)**
Fever	40	22	62	91.81
Generalized weakness	35	20	55	80.88
Bone pain	24	15	39	57.35
Loss of appetite and weight	14	13	27	39.71
P/R bleed	12	3	15	22.06
Epistaxis	9	5	14	20.59
Breathlessness	10	3	13	19.19
Gum bleeding	11	0	11	16.18
Ecchymosis	4	5	09	13.24
Convulsions	1	1	02	2.94
Pallor	42	22	64	94.19
Splenomegaly	34	21	55	80.88
Hepatomegaly	34	18	52	76.47
Lymphadenopathy	5	9	14	20.59

**AML:** Acute myeloid leukemia, **ALL:** Acute lymphoblastic leukemia, **P/R:** Peripheral.

**Table 3:** Mean values of hematological parameters in cases of acute leukemia

**Hematological parameters**	**AML**	**ALL**	**AL**
Hb (g%)	7.09±2.17	7.53±3.03	7.25±2.51
TLC (/mm3)	97317.06±81204.11	112326.8±114817.65	102835.35±94387.92
Blast (%)	61±25.72	68.36±12.01	63.76±21.89
Platelet count (/mm3)	36674.41±27766.64	45480±25314.88	39911.76±27039.901
Neutrophils (%)	9.21±9.104	4.84±4.36	7.6±7.95
Lymphocytes (%)	7.67±6.22	22.2±9.19	13.01±10.21
Eosinophils (%)	1.51±2.26	1.12±1.16	1.37±1.93
Monocytes (%)	1.53±3.54	0.48±1.29	1.15±2.95
Basophils (%)	-	-	-
Band forms (%)	1.3±2.55	0.48±0.96	1±2.13
Metamyelocytes (%)	2.42±3.92	0.6±2.02	1.75±3.44
Myelocytes (%)	3.91±6.44	1.28±5.08	2.94±6.07
Promyelocytes (%)	20.78±19.08	0.72±2.87	20.01±15.74
Promonocytes (%)	23±0.0	0±0.0	23±0.0

**AML:** Acute myeloid leukemia, **ALL:** Acute lymphoblastic leukemia, **Hb:** Hemoglobin, **TLC:** Total leukocyte count.

**Table 4:** Immunophenotyping findings in cases of acute leukemias

**Type of Acute Leukemia**	**Positive markers**				
	**Non-specific lineage markers**	**Lymphoid T-cell markers**	**Lymphoid B-cell markers**	**Myelomonocytic markers**	**Erythroid markers**
**ALL**					
**1) T-ALL**					
1a) T-ALL NOS	Tdt (- to dim +)	CD2 (dim to mod+), CD3, cCD3, CD5, CD7 positive	CD10 (aberrant+)	-	-
1b) T-ALL NK	CD45	CD2, CD7, CD56 (heterogenous+)	-	-	-
**2) B-ALL**					
2a) B-ALL NOS	HLADR, Tdt, CD34 positive, CD34/HLADR co-expression positive	CD7 (aberrant+), CD56 (aberrant+)	CD10+, CD19+, CD10/19 co-expression+, CD20+, CD22+, cCD79a+, CD38+	CD33 (aberrant+)	-
2b) B-ALL RGA					
(i) B-ALL with t(12;21)(p13;q22)-TEL AML1(ETV6 RUNX1)	CD34+	-	CD10+, CD19+	CD13+, CD33+	-
(ii) B-ALL with t(9;22)(q34;q11.2)-BCR ABL1	HLADR+	CD25+	CD10+, CD19+	CD13+	-
**AML**					
**1) AML - RGA**					
1a) AML with t(8;21)(q22;q22.1) RUNX1 RUNX1T1	HLADR+, Tdt (weak+), CD34+	-	-	CD13+, CD33+, CD117+, cMPO+, CD15+	-
1b) APL with PML RARA	CD45+	CD2 (aberrant+)	-	CD13+, CD33+, CD117+, CD64 (mod+), CD11c (- to weak+), cMPO+, CD14 (weak+), CD15 (weak+)	-
1c) AML with mutated NPM1	-	-	-	CD13+, CD33+, CD117+, CD11b+, cMPO+	-
1d) AML with biallelic mutations of CEBPA	HLADR (dim+), CD45+	-	CD38+	CD13+, CD33+, CD64+, CD11b+, CD14+	-
**2) AML–MRC**	CD34+	CD5 (aberrant+), CD7 (aberrant+), CD56 (aberrant+)	-	CD33+, CD117+, CD64+, CD14+	-
**3) AML – NOS**					
3a) AML with minimal differentiation	HLADR+, CD34+, CD34/HLADR co-expression+	CD7 (aberrant+)	CD38 (aberrant+)	CD13+, CD33+, CD117+, cMPO+	-
3b) AML without maturation	HLADR+, CD34+	CD7 (aberrant+)	-	CD13+, CD33+, CD117+, cMPO+	-
3c) AML with maturation	HLADR+, CD34+, CD45+	CD7 (aberrant+), CD56 (aberrant+)	-	CD13+, CD33+, CD117+, CD64+, cMPO+, CD15+	-
3e) Acute monoblastic leukemia	HLADR (dim+), CD45+	CD7 (aberrant+)	-	CD13+, CD33+, CD117+, CD64+, CD11b+, CD11c+, cMPO+, CD14+, CD15+	-
3d) Acute myelomonocytic leukemia	CD45+	CD7 (aberrant+)	-	CD13+, CD33+, CD64+, CD11b+, CD11c+, cMPO+, CD14+, CD15+	-
3f) Pure erythroid leukemia		CD3+	-	CD117+	CD71 (Glycophorin A)+, CD36 (early erythroid marker)+, E-cadherin+

**AML:** Acute myeloid leukemia, **ALL:** Acute lymphoblastic leukemia, **NOS:** Not otherwise specified, **NK:** Natural killer cell, **RGA:** Recurrent genetic abnormality, **MRC:** Myelodysplasia-related changes, **APL:** Acute promyelocytic leukemia, **CD:** Cluster differentiation, **cMPO:** Cytoplasmic myeloperoxidase.

**Table 5:** Distribution of acute leukemia cases according to prognosis

**Type of Acute Leukemia**	**Favorable prognosis (n=41)**	**Unfavorable prognosis (n=27)**	**Mortality** **(n=31)**
**ALL**			
**1) T-ALL**			
1a) T-ALL NOS	1	4	4
1b) T-ALL NK	-	1	1
**2) B-ALL**			
2a) B-ALL NOS	8	7 (aberrant marker positivity, CNS involvement, presence of minimal residual disease after therapy)	7
2b) B-ALL RGA			
(i) B-ALL with t(12;21)(p13;q22)-TEL AML1(ETV6 RUNX1)	3	-	0
(ii) B-ALL with t(9;22)(q34;q11.2)-BCR ABL1	-	1	1
**AML**			
**1) AML - RGA**			
1a) AML with t(8;21)(q22;q22.1) RUNX1 RUNX1T1	6	1 (younger age, TB)	-
1b) APL with PML RARA	6	4 (younger age, DIC, CNS involvement and hypergranular variant)	4
1c) AML with mutated NPM1	1	1 (very old age)	1
1d) AML with biallelic mutations of CEBPA	1	0	-
**2) AML – MRC**	-	2	2
**3) AML – NOS**			
3a) AML with minimal differentiation	-	2	2
3d) Acute myelomonocytic leukemia	3	0	1
3e) Acute monoblastic leukemia	3	1	1
3f) Pure erythroid leukemia	-	1	1
3g) AML without Maturation	-	2	2
3h) AML with maturation	9	-	4

**AML:** Acute myeloid leukemia, **ALL:** Acute lymphoblastic leukemia, **NOS:** Not otherwise specified, **NK:** Natural killer cell, **RGA:** Recurrent genetic abnormality, **MRC:** Myelodysplasia-related changes, **APL:** Acute promyelocytic leukemia.

## DISCUSSION

The present study was conducted to diagnose and classify AL cases according to the WHO (2016) Classification and study their clinicopathological profiles (9). Sixty-eight AL cases were studied for a period of 2 years. AML was more commonly diagnosed than ALL, similar to previous studies (5,6). ALL was found to be more common in children (aged 1-14 years) and AML in adults (aged >30 years) in concordance with the findings of Dores et al. and others (7,12–17). The presenting signs and symptoms also found resonance with these studies (12–17). Fever was the most common complaint in both the leukemias, similar to Kumar et al., Ghosh et al., and others (72-89%) (12–16). This was most likely associated with granulocytopenia and concomitant infections (17,18). Generalized weakness and bleeding manifestations were also similar to these studies (12–16). Gingival bleeding was not seen in any of the ALL cases but was present in AML cases, akin to the studies by Ghosh et al. (23%), Preethi CR (25%), and Sultan et al. (22%). This is suggested to be due to thrombocytopenia and neutropenia (14–16). The most common presenting sign in both ALL and AML was pallor followed by hepatosplenomegaly, similar to the above-mentioned studies, likely related to anemia and organ infiltration, respectively (12–16). The increased peripheral smear blast cell % (68% in ALL and 61% in AML) also mirrors that seen by Kumar et al. (85% in ALL) and Ghosh et al. (57.6% in AML). This correlates to overproduction and blood infiltration of immature white blood cells (12–14).

### Acute Lymphoblastic Leukemia (ALL)

B-cell ALL (B-ALL) was found to be more common than T-cell ALL (T-ALL), in agreement with the prevalence noted by Kumar et al. (63%) (12). Most B-ALL blasts showed CD10 and CD19 positivity. B-ALL not otherwise specified (NOS) revealed CD56 aberrancy, a normal karyotype, and posterior reversible encephalopathy syndrome, with death reported within 5 days of admission. This is in agreement with the findings of Swerdlow et al. and Arber et al. who stated that cranial nervous system (CNS) involvement leads to an adverse prognosis in ALL patients (9,19). B-ALL cases with recurrent genetic abnormality (RGA) t(12;21)(p13;q22) TEL-AML1 (ETV6-RUNX1) revealed blast cell positivity for CD19, CD10, CD34, and CD13 but were typically negative for CD20, CD9, CD66c. These patients achieved molecular remission with a cure rate of >90% (9,19,20). B-ALL RGA (9;22) BCR-ABL1 showed a higher incidence in adults with the blasts cells typically showing high-frequency expression of CD25. Despite imatinib therapy, mortality was recorded within 3 months from diagnosis. They are usually associated with the worst prognosis among all B-ALL RGA cases (9,19). T-ALL cases revealed CD2 and CD7 positivity. T-ALL natural killer cell leukemia (T-ALL NK), diagnosed in a 50-year old male, had an immunophenotypic picture showing CD45 bright+ as well as cytoplasmic myeloperoxidase (cMPO)-negative blasts and marked leukocytosis with 85-90% large-sized lymphoid cells (having high N:C ratio, clumped chromatin and cytoplasmic granules with 1-2 prominent gated nucleoli). Death was recorded within a month from diagnosis. T-LL NOS also presented with mostly an unfavorable prognosis. Other studies have also found T-ALL NK to have an extremely poor prognosis (9,20,21).

### Acute Myeloid Leukemia (AML)

The present study found* *AML-NOS to be the most common type of AML, unlike Nunes et al. who found AML-RGA to be the most prevalent. However, the distribution of AML-RGA subtypes mirrored the works of Nunes et al. (22). AML-NOS was a diagnosis of exclusion, made for cases that cytogenetically did not fulfill the criteria for other AML subtypes, as per the WHO classification (9). However, cytogenetic studies were not performed by Ghosh et al. and Preethi CR, and instead the FAB classification (1976) was employed (8). They found that a small percentage of cases remained, and these were categorized under AML M3 as per FAB classification, which is now categorized as acute promyelocytic leukemia **(**APL) with PML/RARA under AML-RGA (8,14,15). It was noted that the WHO (2017) (9) AML-NOS subcategory closely matched the FAB (1976) AML with maturation (FAB M2) subcategory (8,14,15).* *The presence of APL with PML-RARA, with concomitant disseminated intravascular coagulation (DIC) and CNS involvement, typically led to a very poor prognosis (9,19,23). An increase in promyelocytes on peripheral smear and bone marrow aspirate myelogram was seen, along with the absence of megakaryocytes. Hypergranular variants had multiple Auer rods in the cytoplasm of myeloblasts and/or progranulocytes. Cases of AML-RGA t(8;21)(q22;q22)RUNx1-RUNX1T1 with tuberculosis (TB) required stopping anthracylines and cyclophosphamide for 3 weeks due to a risk of flaring up of TB, but with chances of increase in mortality. Hence, careful anti-leukemic therapy was continued after starting anti-tubercular therapy as early as possible (9,19,23). AML with mutated NPM1 was diagnosed in older individuals with the blast cells typically negative for CD34. Nucleophosmin (NPM) is a surrogate marker for this gene mutation, seen in about 33% of AML cases, with the morphology resembling acute myelomonocytic/monocytic leukemia and has a good prognosis. AML with biallelic mutations of CCAAT enhancer binding protein alpha (CEBPA) was typically associated with a normal Hb level, normal karyotype, and higher blast cell count with a good response to induction chemotherapy (9,19,24,25). According to Walter et al., AML-NOS patients corresponded to the FAB M0 category, known to have significantly worse outcomes than non-M0 cases, similar to the present study (26).

The present study establishes the WHO (2016) classification as a practically useful system for diagnosing the various types of AL, primarily with a focus of providing targeted therapy. Cytogenetics is one of the most important diagnostic parameters as recurrent genetic aberrations have provided insights into the molecular mechanisms of leukemogenesis. The uniformity in categorizing these diseases, afforded by this classification system, permits the use of immunotherapy such as specific targeting of CD expression especially in patients failing induction therapies. Lately, monoclonal antibody therapy has become a significant component of the treatment protocol, for example the use of Gemtuzumab for CD33-positive AML. Stem cell transplantation is also required in many cases receiving chemotherapy and radiotherapy as it helps to replenish their bone marrow reservoir with normal hematopoietic stem cells (27).

This study has its limitations in being a single-center, cross-sectional study with a limited sample size. Multicentric, prospective studies with larger sample size and longer follow-up periods are encouraged to validate the results.

In conclusion, the application of revised 4th edition WHO 2016 classification of acute leukemias confers uniformity in reporting of acute leukemia cases, which aids in treatment by using targeted therapies and helps in prediction of prognosis. The categorization of acute leukemia cases in favorable and unfavorable prognosis groups tells us about the future outcome of cases, and findings of this study show that the unfavorable prognosis group has a dismal prognosis. Overall, the revised 4th edition WHO 2016 classification is very objective, therapy oriented and the need of the hour.

## Conflict of INTEREST

The authors declare no conflict of interest.

## FUNDING

The study was not funded by any government or private funding bodies.
